# Quantitative 3D analysis of huge nanoparticle assemblies†
†Electronic supplementary information (ESI) available.CCDC 1417516–1417520 contain the supplementary crystallographic data for this paper. For ESI and crystallographic data in CIF or other electronic format see DOI: 10.1039/c5nr06962a



**DOI:** 10.1039/c5nr06962a

**Published:** 2015-11-26

**Authors:** Daniele Zanaga, Folkert Bleichrodt, Thomas Altantzis, Naomi Winckelmans, Willem Jan Palenstijn, Jan Sijbers, Bart de Nijs, Marijn A. van Huis, Ana Sánchez-Iglesias, Luis M. Liz-Marzán, Alfons van Blaaderen, K. Joost Batenburg, Sara Bals, Gustaaf Van Tendeloo

**Affiliations:** a EMAT , University of Antwerp , Groenenborgerlaan 171 , B-2020 Antwerp , Belgium . Email: sara.bals@uantwerpen.be; b Centrum Wiskunde & Informatica , Science Park 123 , NL-1098XG Amsterdam , The Netherlands; c iMinds-Visionlab , University of Antwerp , Universiteitsplein 1 , B-2610 Antwerp , Belgium; d Soft Condensed Matter , Debye Institute for Nanomaterials Science , Utrecht University , Princetonplein 5 , 3584 CC Utrecht , The Netherlands; e Bionanoplasmonics Laboratory , CIC biomaGUNE , Paseo de Miramón 182 , 20009 Donostia – San Sebastián , Spain

## Abstract

Nanoparticle assemblies can be investigated in 3 dimensions using electron tomography. However, it is not straightforward to obtain quantitative information such as the number of particles or their relative position. This becomes particularly difficult when the number of particles increases. We propose a novel approach in which prior information on the shape of the individual particles is exploited. It improves the quality of the reconstruction of these complex assemblies significantly. Moreover, this quantitative Sparse Sphere Reconstruction approach yields directly the number of particles and their position as an output of the reconstruction technique, enabling a detailed 3D analysis of assemblies with as many as 10 000 particles. The approach can also be used to reconstruct objects based on a very limited number of projections, which opens up possibilities to investigate beam sensitive assemblies where previous reconstructions with the available electron tomography techniques failed.

## Abstract 2

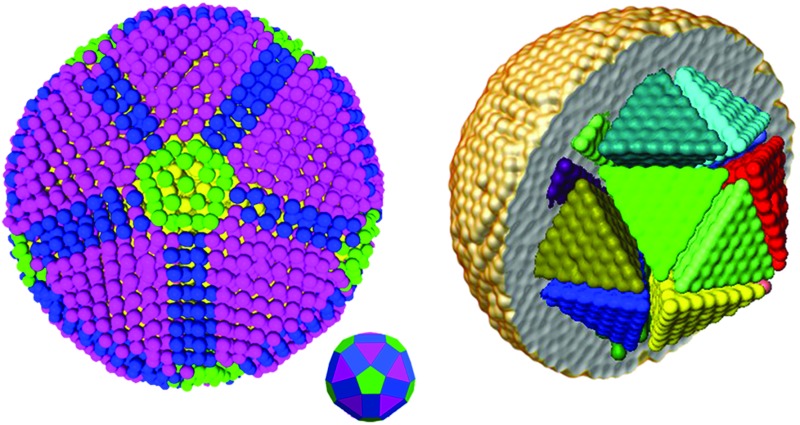
A new reconstruction approach for electron tomography is proposed, enabling a detailed 3D analysis of assemblies with as many as 10 000 particles.

## Introduction

Nanoparticle assemblies attract increasing interest because of the possibility of tuning their properties by adjusting the overall size and shape, the stacking of the individual nanoparticles, and the distances between them.^1–5^ However, as the synthetized systems become more complex, an accurate characterization of the structure becomes more demanding.

Transmission electron microscopy is an important technique to characterize materials at the nanometer scale and below.^6–10^ However, it conventionally only allows for the acquisition of two-dimensional (2D) projections of three-dimensional (3D) objects, which is not sufficient for a quantitative characterization of complex 3D nanostructures. Electron tomography has therefore been developed to overcome this strict limitation, acquiring 2D projections over a large tilt range and combining them through a mathematical reconstruction algorithm.^11^

Electron tomography is a versatile and powerful tool that has been increasingly used in the field of materials science.^12^ It can be used to investigate the morphology and structure of a broad variety of nanomaterials.^13,14^ Tomography has furthermore been combined with spectroscopic techniques such as Electron Energy Loss Spectroscopy (EELS) and Energy Dispersive X-Ray spectroscopy (EDX) for the 3D investigation of chemical composition,^15–18^ bonding nature^19^ and surface plasmons^20^ of nanomaterials. Great effort has also been made in the development of advanced reconstruction algorithms,^21^ enabling quantification of the 3D results^22–24^ and pushing the resolution of the technique to the atomic scale.^25–27^

Also for the characterization of nano-assemblies, electron tomography is nowadays a standard technique,^28–32^ yielding a 3D description of the morphology and inner structure. In comparison to diffraction techniques such as Small Angle X-ray Scattering,^33,34^ one of the main advantages of electron tomography is that the technique also enables a detailed description of non-periodic features such as defects and surface morphology. A quantitative description of the position of the individual nanoparticles furthermore allows a comparison with theoretical models and a better understanding of the mechanisms which rule the self-assembling process.^35–37^

Despite the valuable information that can be obtained by electron tomography, 3D reconstructions based on classical algorithms, such as Weighted Back-projection (WBP)^38^ and the Simultaneous Iterative Reconstruction Technique (SIRT),^39^ suffer from a number of restrictions. The narrow space between the objective lens pole pieces restricts the tilt range of the sample to typically –80° to +80°. This causes “missing wedge artifacts”, which can be observed as an elongation along the beam direction in the final reconstruction.^40^ Furthermore, degradation of the sample due to the electron dose often occurs. As a consequence, the projections are mostly acquired with tilt increments of 1°–5°, yielding an under-sampling of the higher frequencies and a consequent degradation of the resolution with a blurring of the sharper features.^11^

For relatively small assemblies of closed-packed nanoparticles (consisting of 100 particles or less), the number of particles can be determined and their coordinates can be estimated manually.^41^ However, if the number of particles increases and the distance between them is less than the 3D resolution of the tomography experiment,^42^ manual segmentation becomes subjective and quantification of the data is impossible.

Here, we present a novel approach that enables us to determine the coordinates of each nanoparticle in an assembly, even when the assembly consists of up to 10 000 (spherical) particles. This technique will have a major impact as it enables a straightforward quantification of inter-particle distances and 3D symmetry of the stacking. Furthermore, the outcome of these measurements can be used as an input for modeling studies that predict the final 3D structure as a function of the parameters used during the synthesis.^35^

## Results and discussion

### Reconstruction algorithms principles

**Fig. 1:**
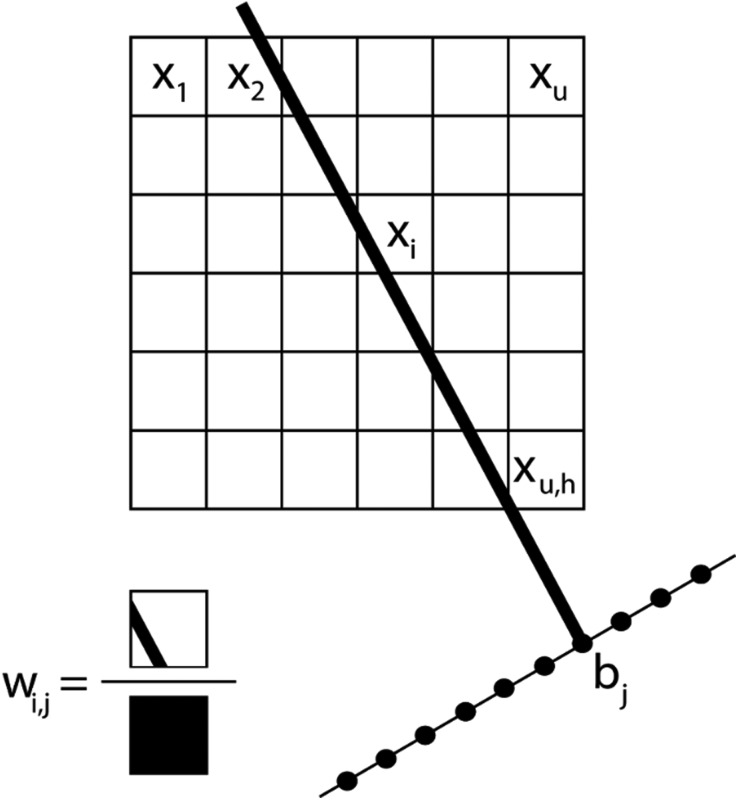
Schematic representation of the formulation of an algebraic reconstruction problem.

In a tomography reconstruction problem, the unknown object is discretized on a grid of pixels, of width *u* and height *h*. Every pixel value is labeled as an unknown *x*_*i*_ for *i* = 1,2,…, *u* × *h*. Probe rays travelling through the object give rise to a projection *b*_*j*_ that equals the sum of the intensities of the probed pixels, each weighted by a coefficient *w*_*ij*_ given by the area covered by the ray traversing that pixel (Fig. 1), such that 
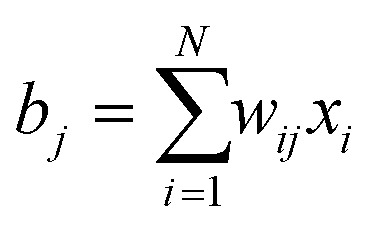
.

The system of linear equations representing the tomographic problem can be written in matrix notation, obtaining eqn (1):

1 *Wx* = *b*

This system of equations is usually underdetermined due to the limited number of projections, leading to an ill-posed inverse problem. To deal with noise in the projection data, the system is typically solved in a least squares sense, minimizing ‖*Wx* – *b*‖_2_. The addition of prior information,^43,44^ or penalty functions^23,24,45^ can be used as strategies to obtain a less-underdetermined reconstruction problem. However, there are a number of samples for which none of the current advanced approaches work well since blurring always occurs^11^ and the missing wedge leads to a superposition of the particle boundaries, hampering to distinguish them. This is particularly the case for large assemblies of spherical (or nearly spherical) particles or when only a limited number of projections are available. It must be noted that during the 3D investigation of an assembly of nanoparticles, the exact shape of the individual particles is often not of crucial interest and we can assume that they correspond to perfect spheres. If the size of the particles can be estimated, we can use discrete spheres as basis elements and the problem is reduced to the reconstruction of the center coordinates of these spheres. Now we can use the following image transformation:

2 *x* = *Cy*

where *y*_*i*_ = 1 if a sphere center is located at pixel *x*_*i*_. *C* corresponds to a matrix that transforms the centers into pixelized images of spheres. Our tomography problem (eqn (1)) then becomes:

3 *WCy* = *b*

Since even for large assemblies, the number of particles is small compared to the number of voxels, the coefficient vector *y* will be very sparse. To incorporate this sparsity assumption in the reconstruction, we solve the following problem:4
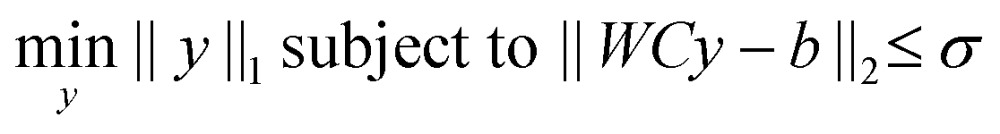
where *σ* is the noise level. While the tomography problem of eqn (1) typically does not have a unique solution, computing a solution of eqn (4) instead leads to a solution that contains relatively few nonzero pixel values, corresponding to a sparse assembly of spheres. This concept is mathematically explained in the ESI† and the implementation is presented in the Experimental section. In the remainder, we will refer to this approach as Sparse Sphere Reconstruction (SSR).

### Quantitative electron tomography of nanoparticle assemblies

To demonstrate the power of SSR, two examples are presented. In the first example, close-packed assemblies of cobalt iron oxide nanoparticles with a diameter of about 9 nm are presented. The spherical assemblies have a diameter up to 300 nm and can contain more than 9000 particles. Typically, 3D characterization of such an assembly is highly challenging.

The second example shows how SSR can be used for an accurate reconstruction of assemblies even with a minimum number of projections, enabling the investigation of beam sensitive assemblies.

**Fig. 2:**
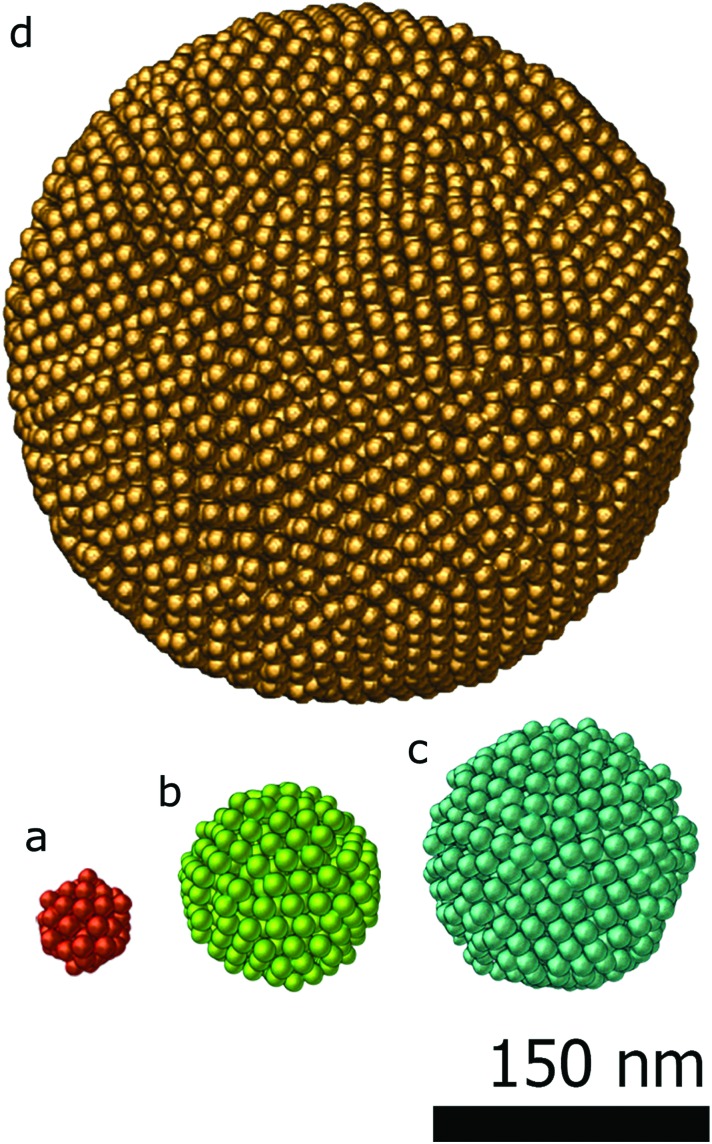
SSR reconstructions of Fe–Co–O nanoparticles assemblies with different size: (a) 50 nm diameter containing 70 particles. (b) 100 nm diameter containing 574 particles. (c) 150 nm diameter containing 1305 particles. The icosahedral symmetry of the particle is clear from this view. (d) 300 nm diameter containing 9301 particles.

In the first study, the investigated assemblies (Fig. 2) have an increasing diameter and number of particles. The assemblies diameters are approximately 50 nm, 100 nm, 150 nm and 300 nm and contain 70, 574, 1305 and 9301 particles respectively. The reconstructions obtained through the proposed approach (SSR) are presented in Fig. 2. For comparison, the SIRT equivalent (same magnification, same point of view) of the figure is presented in the ESI (Fig. S1†).

**Fig. 3:**
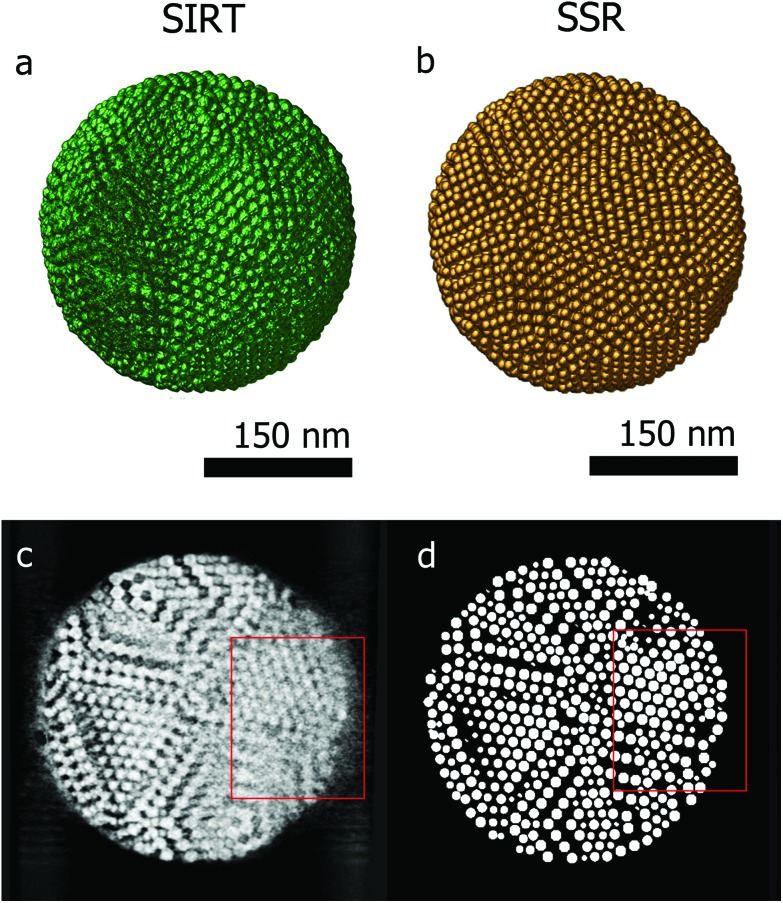
(a) 3D visualization of the SIRT reconstruction of an assembly of about 9000 Co–Fe–O particles. (c) Orthoslice acquired through the SIRT reconstruction, missing wedge artifacts are highlighted by the red rectangle. (b, d) 3D visualization and orthoslice of the corresponding SSR reconstruction.

Fig. 3a presents the SIRT reconstruction of the larger assembly of Co–Fe–O particles and provides a reasonable qualitative description of the shape and size of the assembly, however any quantitative analysis is hampered by the (missing wedge) artifacts and the poor resolution. Indeed, the information along the direction most affected by the missing wedge (red rectangle Fig. 3c) is insufficient to enable a manual segmentation. Fig. 3d presents an orthoslice through the SSR reconstruction at the same position as in Fig. 3c. It is clear that information, lost because of the missing wedge, is recovered by using the SSR reconstruction. This can be better observed for the smaller assemblies, for which projections were acquired over smaller tilt ranges (relative to the larger assembly – details in the Experimental section) causing more severe missing wedge artifacts.

**Fig. 4:**
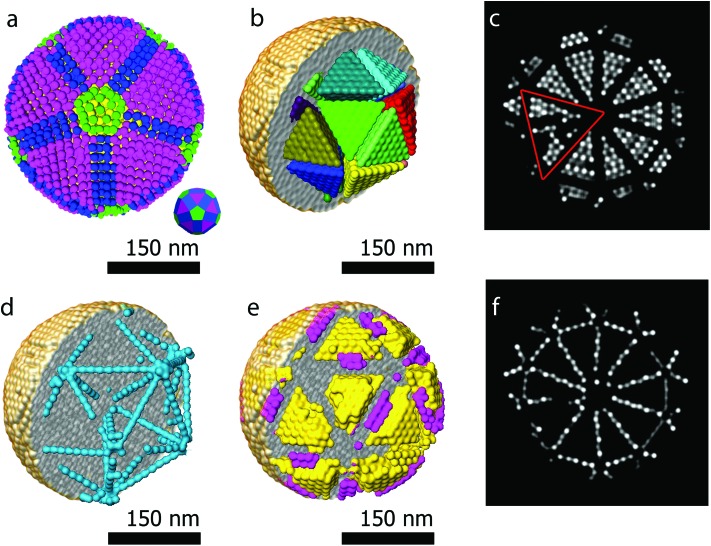
(a) 3D visualization of the rhombicosidodecahedral outer structure. (b) Icosahedral core consisting of 20 tetrahedra with particles in an fcc stacking (different colors are used to highlight separated tetrahedra and improve the visualization of the 3D structure). (c) Orthoslice through the reconstruction showing only the particles in an fcc stacking. The red triangle highlights a defect in two tetrahedra, which causes a deformation of the Mackay icosahedral core. (d) Particles with icosahedral packing, the tetrahedra visualized by the blue particles are arranged in five-fold symmetry. (e) fcc stacked particles composing part of the outer shell (different colors are used to highlight separated fcc clusters on the surface and improve the visualization of the 3D structure). (f) Orthoslice through a 3D visualization of the particles forming the twin planes (hcp stacking).

Another major advantage of our approach is that the number of particles and the coordinates are a direct output of the reconstruction without the need for segmentation. From our reconstruction, it can be deduced that the outer morphology of the assembly corresponds to a rhombicosidodecahedron (Fig. 4a). To investigate the crystallographic nature of the stacking of the nanoparticles, local bond order parameters were calculated^46,47^ and clustered through a *k*-means algorithm.^48^ A careful analysis reveals that the assembly core corresponds to an icosahedron consisting of 20 tetrahedra (Fig. 4b). The particles in the tetrahedra are arranged according to a local fcc stacking and form a Mackay icosahedron.^49^ The tetrahedra are separated from each other and are arranged with five-fold symmetry (Fig. 4b and d). Twinning planes are also found between the tetrahedra and the outer shell of the assembly which yields an hcp stacking. Fig. 4f presents an orthoslice through the reconstruction showing areas with hcp stacking, whereas Fig. 4c shows an orthoslice through areas with fcc stacked particles. The outer shell is mostly composed by particles in an fcc arrangement (Fig. 4e) forming a surface with anti-Mackay icosahedral termination. Interestingly, Fig. 4d shows all the particles arranged in an icosahedral packing. It can be seen that they extend along the tetrahedral edges highlighting the icosahedral core structure. The five-fold icosahedral symmetry has been shown to be the most favorable geometry in short-range ordered clusters composed of particles with attractive interactions and is found in many systems.^50,51^ It is a relatively new finding that icosahedral ordering is the equilibrium structure also for hard particles, for which entropy is the only deciding contribution to the free energy, that are made to self-assemble in a spherical confinement.^37^ Assemblies up to 700 particles are expected to carry an icosahedral structure, which changes to a rhombicosidodecahedral symmetry between 700 and 70 000 particles and finally pure bulk fcc for more than 70 000 particles.^37^ Here, with 9300 particles, the structure observed is a rhombicosidodecahedron presenting an inner distorted Mackay icosahedron, in perfect agreement with de Nijs *et al.* ^37^ The distortion of the icosahedron is caused by defects in some of the tetrahedra. This can be observed in Fig. 4c where, an orthoslice through the structure shows two of the tetrahedra (red triangle) affected by defects that alter the perfect tetrahedral shape, causing general inhomogeneity in the size of all the other tetrahedra (this can be better observed in the online interactive visualization available at ; http://ematweb.uantwerpen.be/colouratoms/jsc3D/demos/FeCoO_Assembly_300nm.html).

**Fig. 5:**
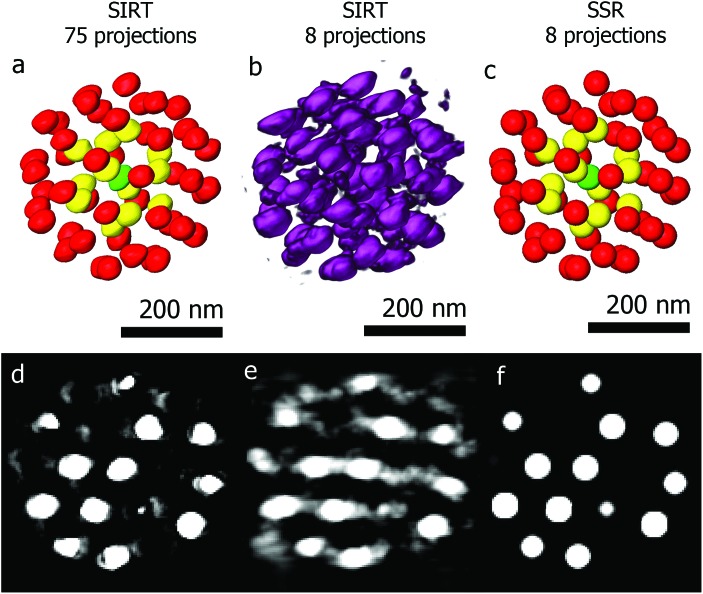
(a, d) 3D visualization of a SIRT reconstruction of an assembly of Au spheres (75 projections) and an orthoslice acquired through the reconstruction. (b, e) 3D visualization and orthoslice through the SIRT reconstruction of the same assembly where only 8 projections were used. (c, f) 3D visualization and orthoslice through the SSR reconstruction of the same assembly where only 8 projections were used.

The second example is a less complex structure consisting of an assembly of gold particles embedded in a polystyrene matrix. We will demonstrate the power of using SSR when reconstructing beam sensitive assemblies. Long acquisition times form a main drawback of tomography, limiting the technique to beam resistant samples. Reducing the angular sampling frequency reduces the electron dose, but also deteriorates the quality of the final reconstruction. Fig. 5a shows a 3D visualization of a SIRT reconstruction of an assembly of gold quasi-spherical particles embedded in a polystyrene matrix.^41^ The reconstruction is based on a series of 75 projections (–70° to +78°, 2° tilt increment). Segmentation with different colors is performed to enable a better visualization. Fig. 5b and c show the SIRT and SSR reconstructions based on the same series, but using only 8 projections (–70° to +70°, 20° tilt increment). Although the details about the shape of every individual particle are lost, valuable information concerning the structure (such as inter-particle distances and local symmetries or 3D stacking) is retrieved. The fact that we can reduce the number of projection images by a factor of ten opens up the route to obtain 3D quantitative information for beam sensitive systems.

The approach presented here, is currently limited to assemblies of monodispersed spherical particles. A logical next step is to apply the method to assemblies in which two or more different particle diameters are considered. This can be done by expanding the matrix *C* to contain spheres of various diameters. We are currently working to implement this possibility.

Furthermore, assemblies consisting of anisotropic particles have recently obtained increasing attention as well.^31,32^ However, in the case of particles with more complex shapes (rods, cubes, *etc.*) a different approach has to be adopted. We envisage that a quantitative 3D characterization of such assemblies will become possible by combining the presented method with the possibility of using a pre-defined “dictionary” of particle features (*e.g.* edges, corners, *etc.*). Obviously, as the dictionary grows in size, more projections will be needed. The potential of such a methodology for electron tomography was recently demonstrated albeit for simulated images.^52^

## Experimental

### Implementation of the SSR algorithm

The use of GPUs has become a widespread approach to tackle heavy computational problems.^53^ The recent release of the ASTRA toolbox,^54–56^ an open source, GPU-accelerated library for 3D image reconstruction in tomography, enables the development of custom algorithms and methods in Matlab. We developed the new approach described here, following the method proposed by Bleichrodt *et al.* ^57^ by combining the ASTRA and the SPOT toolboxes^58^ to generate the problem in matrix notation (in Matlab) and then use a general sparse solver SPGL1^59^ for the SSR problem. Technical details of the implementation are presented in the following paragraph.

### Sparse sphere reconstruction (SSR)

Nanoassemblies of spherical particles do not have a sparse representation on a voxel grid, which is used in the linear model in eqn (1). However, we can still employ sparsity promoting linear solvers by using a sparse image representation. If we assume that all particles are perfect spheres (no inhomogeneity on the boundaries) and if the size of the particles can be estimated, we can use discretized spheres as basis elements and encode an image of spheres by the center coordinates of these spheres. This is done by applying the image transformation of eqn (2). (More technical details can be found in the ESI.†)

Note that the matrix *C* could be seen as a convolution operator, but forming the matrix explicitly is not practical due to its size. We can use image convolution of the sphere centers *y* with a discretized sphere of size 2*r* × 2*r* × 2*r* by using one of Matlab's convolution routines such as imfilter (or implementing a convolution in the Fourier domain with fftn). The Spot operator is an object that can be used with Matlab's matrix syntax (*e.g.* to compute *x* = *C* × *y*), but internally it calls the imfilter routine to actually perform this matrix operation. This allows easy implementation of eqn (4) which is simply reduced to:*y* = spgl1(W* *C*,*b*);

Solving eqn (4) represents the first step in obtaining the final reconstruction. This solution should be the sparse representation *y*, but as can be observed from column 2 of Fig. 7, it is still not a perfect grid of ones and zeros representing the centers of the disks. Blurring and striking artifacts due to under-sampling of higher frequencies and missing *wedge also occur. Nevertheless, the sparse representation can be easily recovered and the artifacts eliminated by applying a local maximum extraction routine. To do that, each pixel is compared with its neighbors and selected as a local maximum if the neighbors are indeed smaller than the current pixel value. The neighbors are defined by a neighborhood, which is a small mask of zeros and ones, indicating whether a pixel is in the neighborhood or not. The mask will be the same basis shape used in defining *C*. The second step of the reconstruction will be then carried out through the application of the local maximum extraction in order to remove the artifacts and recover the expected sparse representation *y*. Finally, the operator *C* (eqn (2)) is applied to *y*, in order to obtain the final reconstruction of the object.

**Fig. 6:**
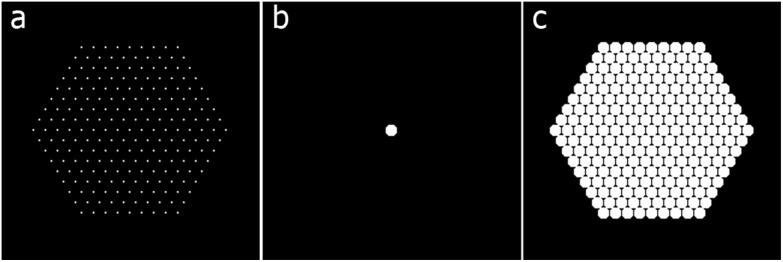
A hexagonal lattice of pixels (a) is convolved with (b) to generate the phantom (c). In the sparse formulation of eqn (2), (c) represents *x*, (b) represents the prior knowledge implemented through the matrix *C* and (a) is the exact solution *y*.

**Fig. 7:**
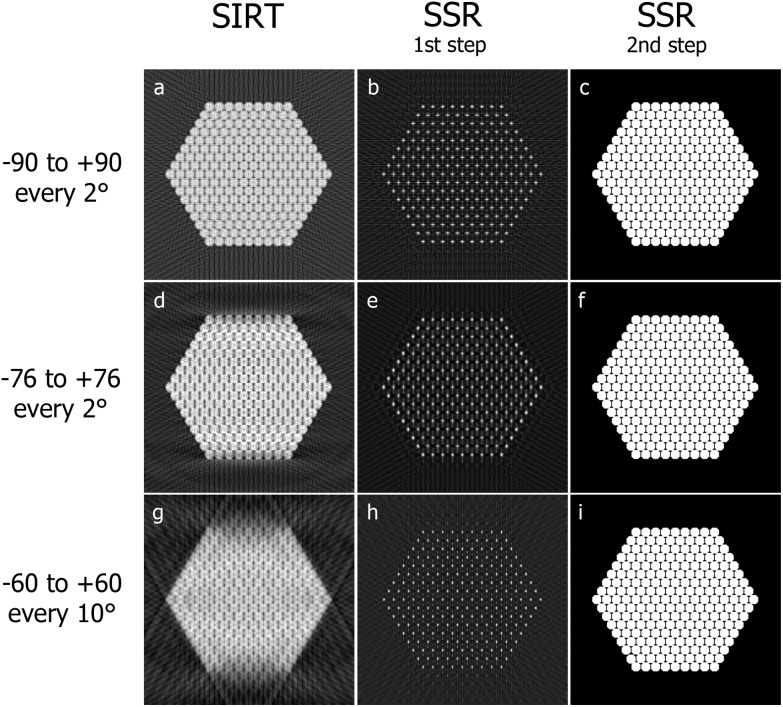
SIRT reconstructions (a, d, g) of the phantom in Fig. 6c for different angular intervals and sampling frequencies. Solution retrieved minimizing ‖*Wcy* – *b*‖_2_ (b, e, h). Finally in (c, f, i) are shown the reconstructions obtained extracting the local maxima from (b, e, h) and convolving them for the prior knowledge shape (Fig. S3b†).

A two-dimensional example of the method is presented. The phantom in Fig. 6c is reconstructed with SIRT and then SSR for different tilt ranges and frequencies (Fig. 7).

### Acquisition and alignment of tomography tilt series

All series were acquired in Scanning Transmission Electron Microscopy (STEM) mode with the use of a High Angle Annular Dark Field detector (HAADF) STEM. For the assembly of Au particles, an FEI Tecnai G2 electron microscope operated at 200 keV was used and for the Co–Fe–O nanoparticles assemblies, the tilt series were acquired using an aberration corrected cubed FEI Titan 60–300 electron microscope, operated at 300 keV. A Fischione 2020 single tilt tomo-holder was used for all the experiments, with the following tilt ranges: –48° to +62° with an increment of 2° for the 50 nm Co–Fe–O assembly, –58° to +76° with an increment of 2° for the 100 nm Co–Fe–O assembly, –32° to +76° with an increment of 2° for the 150 nm Co–Fe–O assembly, –71° to +76° with an increment of 1° for the 300 nm Co–Fe–O assembly and –70° to +78° with an increment of 2° for the Au sample. Projection images from each series are presented in the ESI (Fig. S2 to S6†). Alignment of the tilt series was performed through Matlab routines based on cross-correlation, a centroid alignment method and manually with IMOD.^60^ The series were corrected for cupping artifact as described by Van den Broek *et al.* ^61^ and in case of the Co–Fe–O sample, denoising of the series was performed through the application of a Gaussian filter. We observed that noise reduction at the expense of a loss of resolution in the starting series resulted in a better quality of the final reconstruction.

### Synthesis and TEM preparation of the Fe–Co–O assemblies

The assemblies were synthesized using the emulsion based bottom-up self-organization method.^62^ First, the core–shell FeO/CoFe_2_O_4_ nanocrystals (NCs) were synthesized according to the procedure of Kovalenko *et al.* ^63^ These pre-synthesized NCs are redispersed in cyclohexane. This oil phase containing the NCs is mixed with an aqueous solution containing multiple surfactants (sodium dodecylsulfate, dextrane (mol wt 1 500 000–2 800 000) and distillated water) through vigorous stirring. To make sure that the clusters are monodispersed, the pre-mixed emulsion is pumped into a couette shear mixer.^64^ Next, cyclohexane is evaporated from the sheared emulsion by heating it to 68 °C. Evaporation of cyclohexane from the oil micro-emulsion droplets cause them to shrink, and the rising of NCs concentration promotes their self-assembly into 3D colloidal spheres.

In order to study the assemblies in the electron microscope, TEM grids need to be prepared. However, depositing a solution drop onto the grid and letting it dry at room temperature would cause the assemblies to deform because of the capillary forces between the assemblies and the carbon coated grid. Therefore, the solution is deposited on the TEM grid, immediately vitrified at liquid nitrogen temperature and then sublimated in an Environmental Scanning Electron Microscope (ESEM) at a controlled temperature and pressure. This avoids contact between the colloidal particles composed of the NCs and a drying liquid that is on the outside of the supraparticles, preventing the deformation of the assemblies.

### Synthesis and TEM preparation of the Au assemblies

Colloidal gold superspheres were prepared using a recently reported procedure.^32^ Briefly, gold nanoparticle building blocks (20 nm diameter) were stabilized with a hydrophobic polymer (thiolated polystyrene, molecular weight = 53 000 g mol^–1^). The length of the polymer chain was determined by dynamic light scattering to be 39 nm. The polystyrene-stabilized particles spontaneously self-assembled upon slow addition of water to a dispersion in tetrahydrofuran, and the formed assemblies were subsequently stabilized within polymeric micelles of a di-block copolymer (polystyrene-*block*-poly acrylic acid). The final size of the assemblies in solution was 160 ± 4 nm.^41^ TEM samples were prepared by drop casting the aqueous solution of the assemblies on holey, carbon-coated copper grids.

## Conclusions

We present a new approach for the 3D reconstruction of assemblies of spherical nanoparticles, based on a sparse reformulation of the tomographic problem derived from prior knowledge of the homogenous nature of the objects composing the structure. It is clear that SSR is able to deliver an accurate reconstruction of complex nanoparticle assemblies consisting of up to several thousands of particles. For electron beam sensitive materials, where no extended *tilt series can be obtained, the SSR method still allows one to reconstruct the 3D assembly with a limited number of projections. This methodology opens up the route to a better understanding of the formation of these assemblies as the outcome of these experiments can be used as accurate input models for simulation studies.
