# Supplementary material for: Quantitative 3D analysis of huge nanoparticle assemblies
Source: Nanoscale. 2015 Nov 26;8(1):292–9. doi: 10.1039/c5nr06962a (PMC4819762; doi:10.1039/c5nr06962a)
Supplement: Supplementary file 1 [file NR-008-C5NR06962A-s001.pdf]

Electronic Supplementary Material (ESI) for Nanoscale.
This journal is © The Royal Society of Chemistry 2015
Please do not adjust margins

Journal Name

ARTICLE

Supporting Information

Quantitative 3D analysis of huge nanoparticle assemblies

Daniele Zanaga, Folkert Bleichrodt, Thomas Altantzis, Naomi Winckelmans, Willem Jan Palenstijn, Jan Sijbers, Bart de Nijs, Marijn A. van Huis, Ana Sánchez-Iglesias, Luis M. Liz-Marzán, Alfons van Blaaderen, K. Joost Batenburg, Sara Bals, Gustaaf Van Tendeloo*

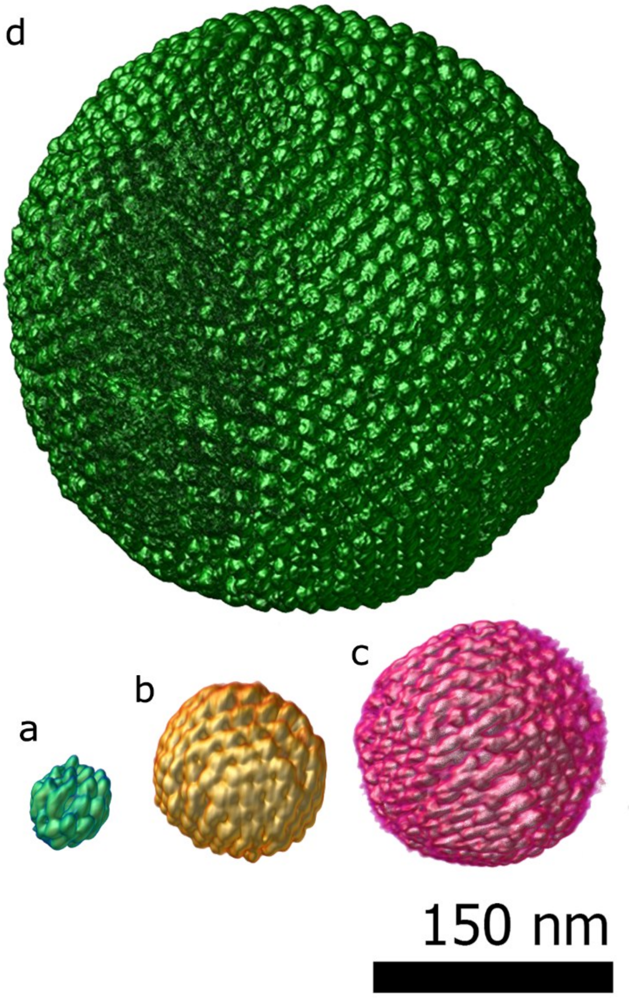

Figure S1: SIRT reconstructions of Fe-Co-O nanoparticles assemblies with different size: (a) 50 nm diameter containing 70 particles. (b) 100 nm diameter containing 574 particles. (c) 150 nm diameter containing 1305 particles. The icosahedral symmetry of the particle is clear from this view. (d) 300 nm diameter containing 9301 particles.

Basis change

The image transformation of Equation 2 is explained here. This transformation allows to reconstruct directly only the centers of every particle, making quantification of number, position and packing of particles, straightforward.

For simplicity we start from the 2D case where we can consider disks instead of spheres, but the extension to 3D is straightforward. Let $d_i = i - r$ indicate the center coordinate of pixel i, then a disk is represented as:

$$b_{ij} = \{1 \; if \; d_i^2 + d_j^2 \le r^2, \quad \text{for i,j = 1, …, 2r}$$
$$\{0 \; otherwise$$

Now we can use the image transformation (Figure S2) of Equation 2, where $y_i = 1$ if a sphere center is located at pixel x_i. Each column $\vec{c}_j$ is a vectorized $u \times h$ image containing one discrete disk with its center at pixel x_j. This matrix C is too large, especially in the 3D case to form it explicitly. However, we can also see it as a convolution operator, where we convolve the disk centers y by the discrete $2r \times 2r$ image of a single disk. This operation can be done efficiently by using Matlab's built-in image convolution method.

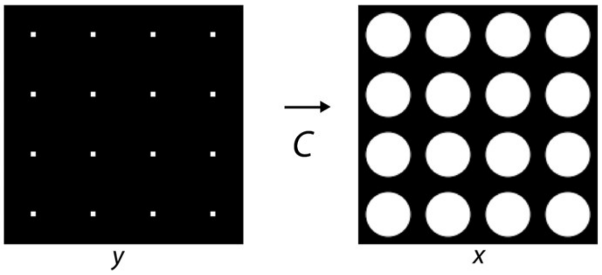

Figure S2: Image transformation of disk centers to the voxel representation.

Please do not adjust margins

Please do not adjust margins

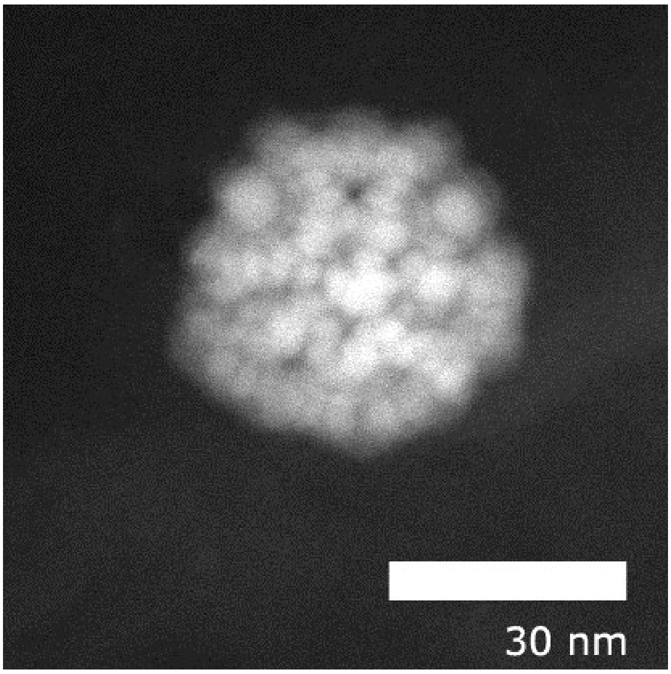

Figure S3: HAADF-STEM projection of the Fe-Co-O assembly with a diameter of approximately 50nm

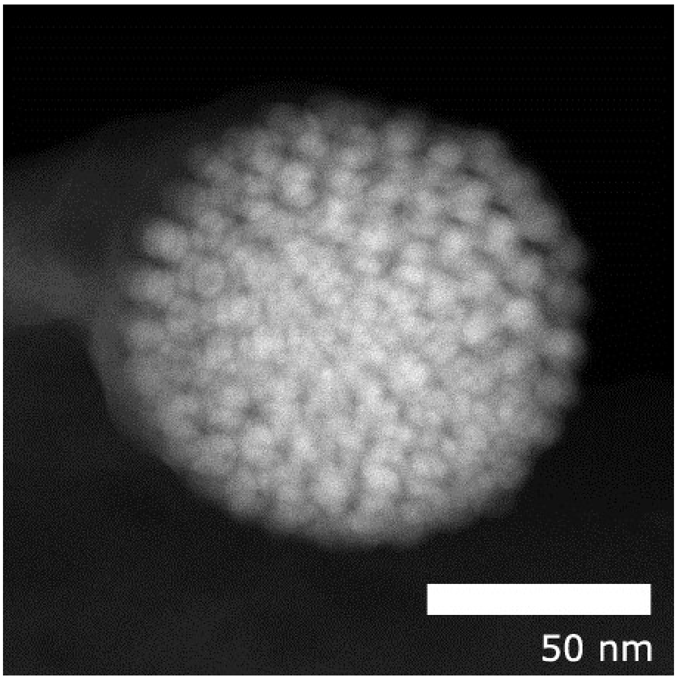

Figure S4: HAADF-STEM projection of the Fe-Co-O assembly with a diameter of approximately 100nm

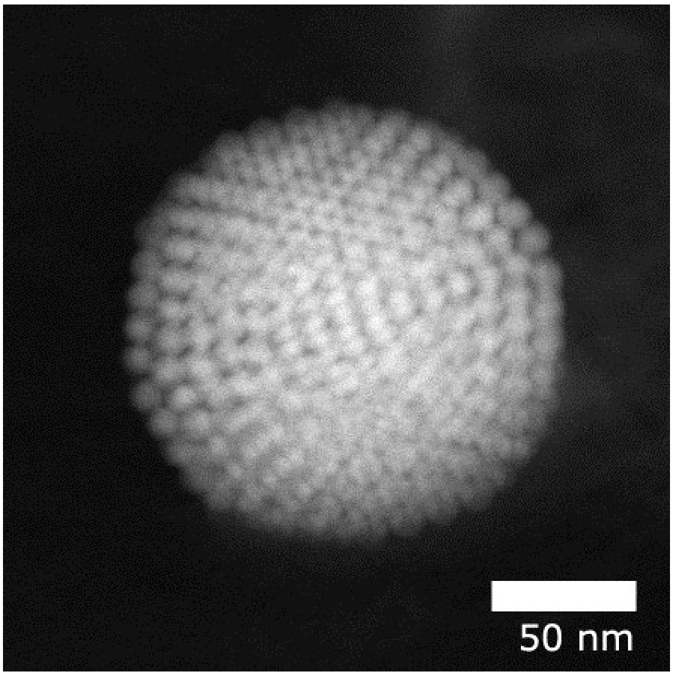

Figure S5: HAADF-STEM projection of the Fe-Co-O assembly with a diameter of approximately 150nm

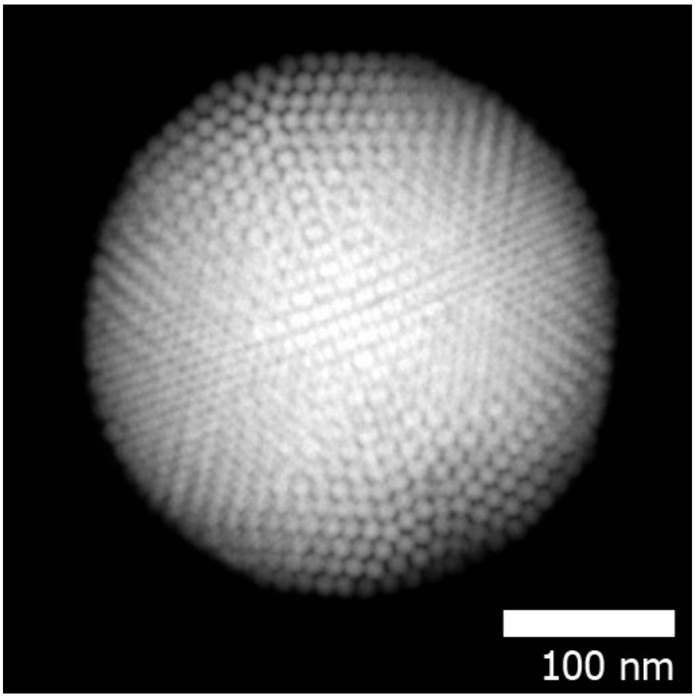

Figure S6: Figure S4: HAADF-STEM projection of the Fe-Co-O assembly with a diameter of approximately 300nm

This journal is © The Royal Society of Chemistry 20xx

Please do not adjust margins

Please do not adjust margins

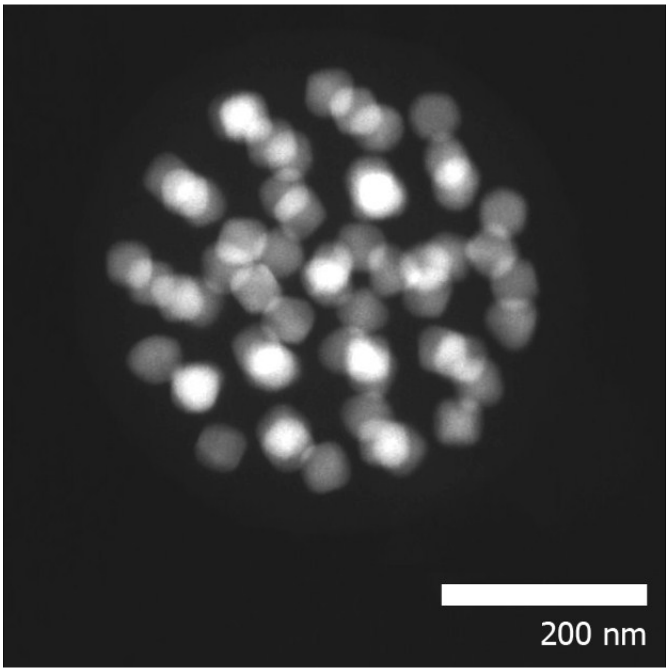

Figure S7: HAADF-STEM projection of the Au particles assembly.

Please do not adjust margins

Please do not adjust margins

This journal is © The Royal Society of Chemistry 20xx

Please do not adjust margins
